# Radicular Pain Management Using Ultrasound-Guided Versus Fluoroscopy-Guided Epidural Steroid Injections: A Systematic Scoping Review of Comparative Studies

**DOI:** 10.7759/cureus.68042

**Published:** 2024-08-28

**Authors:** Wassim Hassan, Ahmad Hassan, Enyo Ablordeppey, Jabra Mustafa, Lauren Yaeger, Ibrahim S Al-Busaidi

**Affiliations:** 1 Anesthesiology, University of Illinois College of Medicine, Chicago, USA; 2 Anesthesiology, Washington University in St. Louis School of Medicine, St. Louis, USA; 3 Anesthesia and Critical Care, Washington University in St. Louis School of Medicine, St. Louis, USA; 4 Radiology, Loyola University Chicago Stritch School of Medicine, Chicago, USA; 5 Library Sciences, Washington University in St. Louis School of Medicine, St. Louis, USA; 6 General Practice, University of Otago, Christchurch, NZL

**Keywords:** epidural steroid injections, anesthesiology, radicular pain, pain management, fluoroscopy, ultrasound-guided, ultrasound

## Abstract

Back pain is the leading cause of disability globally and results in a substantial medical and economic burden. Epidural steroid injections (ESIs) have been widely used as a treatment for back pain with radiculopathy of various etiologies. Ultrasound guidance (UG) for delivering ESIs can reduce costs and facilitate the procedure in resource-limited settings compared to the current standard technique of using fluoroscopic guidance (FG). This scoping review aimed to compare the clinical outcomes between UG and FG ESIs in the treatment of radicular pain. Systematic searches of Embase, Ovid Medline, Scopus, CENTRAL (Cochrane Central Register of Controlled Trials), CDSR (Cochrane Database of Systematic Reviews), and ClinicalTrials.gov were conducted in accordance with PRISMA-ScR (Preferred Reporting Items for Systematic reviews and Meta-Analyses extension for scoping reviews) guidelines. Randomized controlled trials (RCTs) and comparative observational studies investigating the outcomes between UG and FG ESIs in the treatment of radicular pain were included. The risk of bias for included RCTs was assessed using the Cochrane Collaboration risk-of-bias tool. From 1,659 potentially relevant publications, eight studies (five RCTs and three retrospective comparative studies) were included. Five of the studies were conducted in the Republic of Korea, one in China, one in India, and one in Egypt. All studies reported no significant difference between UG and FG ESIs in success rate, pain index, and postoperative disability (p > 0.05). One study reported increased intravascular injections in the FG group, but this did not reach statistical significance (p > 0.05). One study reported decreased needle-placement time in the UG group (p < 0.001). One study reported decreased total operation time in the UG group (p < 0.05). Overall, treatment outcomes and adverse events profile are comparable between UG and FG ESIs for radicular pain. UG ESIs reduce costs, minimize radiation exposure, facilitate vessel identification, prevent injury, and potentially save intraoperative time while offering the same benefits as FG injections. Future studies should focus on long-term outcomes, cost-effectiveness, and the impact of UG ESIs on patient satisfaction and quality of life.

## Introduction and background

Back pain is a ubiquitous and often debilitating medical condition affecting millions worldwide, significantly impacting their quality of life and productivity [1]. It is a leading cause of disability, making it a substantial burden on healthcare systems and economies [2]. The lifetime prevalence of back pain is estimated to be approximately 84% in adults, with around $200 billion spent each year in managing this condition [3]. The etiologies of back pain are numerous and include traumatic, degenerative, oncologic, inflammatory, metabolic, postural, and idiopathic. Determining the etiology of back pain is often a painstaking process that requires a thorough history and physical examination and oftentimes various modalities of imaging [4].

The approach to the treatment of back pain varies depending on the underlying cause, and the physician must first identify the presence of any red flags (e.g., fever, focal tenderness to palpation) to rule out life-threatening emergencies such as infectious or oncologic causes [5]. First-line treatments for back pain are typically non-pharmacologic and include patient education, physical therapy, superficial heat application, acupuncture, massage, and continuation of normal routines while avoiding activities that precipitate pain [6]. Second-line pharmacologic treatment can include nonsteroidal anti-inflammatory drugs (NSAIDs), opioids, and muscle relaxants [7].

Radiculopathy is a common cause of back pain and results when a spinal nerve root is compressed or irritated [8]. The most common cause of radiculopathy is a herniated disc, but regardless of the underlying cause, a cornerstone in the treatment of radicular pain is epidural steroid injections (ESIs) [9]. ESIs are the most performed procedure for pain management in the United States and have been shown to reduce pain, restore function, and delay invasive surgery [10]. ESIs are considered a less risky treatment option compared to opioids, which have the potential for abuse [11].

ESIs induce pain relief primarily through the reduction of inflammation [10]. Corticosteroids inhibit phospholipase A2, preventing the production of pro-inflammatory eicosanoids which can induce pain. It is also hypothesized that ESIs induce pain relief via lavage of the epidural space. A systematic review conducted by Rabinovitch et al. found a positive correlation between larger volumes of fluid injected into the epidural space (irrespective of the injected medication) and greater relief of radicular pain [12].

Delivery of ESIs typically involves fluoroscopic guidance (FG), and less frequently, computerized tomography (CT) to ensure proper needle placement [13]. This has become the standard of care, although recent studies have shown ultrasound-guided (UG) ESIs to be a safe, reliable, and radiation-free alternative to fluoroscopy [14,15]. In recent years, UG ESIs have gained attention for the potential advantages they can provide over FG ESIs, such as the lack of radiation exposure, equipment mobility, and visualization of soft-tissue and real-time needle trajectory. Current research on this topic remains limited. This systematic scoping review aims to summarize the potential advantages that UG ESIs can provide over FG ESIs and to evaluate the current literature on this topic to characterize the evidence regarding the efficacy and safety between these two imaging modalities.

## Review

Methods

We conducted a scoping review following the methodological framework proposed by Arksey and O’Malley [16] and its recent update by Levac et al. [17]. The conduct and reporting of this scoping review were also guided by the Joanna Briggs Institute Methods Manual for Scoping Reviews [18]. This approach ensured a comprehensive exploration of the literature relevant to our research objectives.

This protocol was registered with the Open Science Framework (OSF) (https://osf.io/tjc8h) aligning with established transparency practices in scoping review methodology. The PRISMA (Preferred Reporting Items for Systematic Reviews and Meta-Analyses) extension for scoping reviews (PRISMA-ScR) was used to guide our reporting [19].

Eligibility Criteria

Eligible studies were included based on the following criteria: (1) randomized controlled trials (RCTs) or comparative observational studies investigating the outcomes between UG FG ESIs in the treatment of radicular pain; (2) study population consisting of patients 18 years old or older with radicular back pain; and (3) English-language publications. Exclusion criteria were as follows: (1) studies other than RCTs and comparative observational studies; (2) studies in which ultrasound-guided steroid injection was not compared to fluoroscopy-guided injection; (3) studies that did not report a primary outcome; and (4) non-English transitions to studies.

Data Sources and Study Identification

A specialist medical librarian (LHY) searched article databases and clinical trial registers from inception for relevant records including the concepts of UG or FG and radicular pain and epidural injection. The librarian created systematic search strategies using a combination of keywords and controlled vocabulary in Embase 1947-present, Ovid Medline 1946-present, Scopus 1823-present, Cochrane Central Register of Controlled Trials (CENTRAL), Cochrane Database of Systematic Reviews (CDSR), and Clinicaltrials.gov 1997-present. All search strategies were completed on April 13, 2022, with no added limits, and a total of 2,127 results were found. In all, 880 duplicate records were deleted after using the de-duplication processes described in Bramer et al. [20] and one additional duplicate was removed by Covidence.org resulting in a total of 1,246 unique citations included in the project library. The searches were updated on January 2, 2024, by re-running each search from database inception and de-duplicating the total results in Covidence.org to find 286 new results. Fully reproducible search strategies for each database can be found in Appendix A.Updated search dates and results are included with the full searches.

Study Selection

All records identified in the search were imported into Covidence.org. Two reviewers (AH and WH) independently assessed the identified citations for eligibility based on the title and abstract. The abstracts included were subsequently screened in full text according to the inclusion and exclusion criteria. Differing opinions were resolved through discussion or a third author’s judgment (ISA). Reference lists of identified full-text articles were searched for additional studies.

Data Extraction

Data from included records were extracted into a Microsoft Excel, version 16 (Microsoft Corporation, Redmond, WA, USA) spreadsheet. The reviewers collected relevant characteristics from each study, including author, publication date, country, number of patients, demographic characteristics, severity of diabetic foot, and reported outcome measures.

Quality Appraisal/Risk-of-Bias Assessment

Two reviewers (AH and WH) independently assessed the quality of each included study using the Cochrane Collaboration tool for assessing risk of bias [21], assessing the following domains: selection bias, performance bias, detection bias, attrition bias, reporting bias, and other biases. The Newcastle-Ottawa Scale was used to assess the risk of bias in observational studies [22,23].

Data Analysis

Due to the heterogeneity in the methods and outcome measures of the included studies, statistical comparisons were not feasible. Consequently, a narrative description of the data was chosen as an appropriate method to achieve this study’s aims. This methodology is commonly employed in evidence-based journals and databases [24,25]. The authors systematically analyzed all reported outcomes from the included studies. They performed a thematic analysis and reported only those findings that aligned with the objectives of the work.

Results

Literature Search

**Figure 1:**
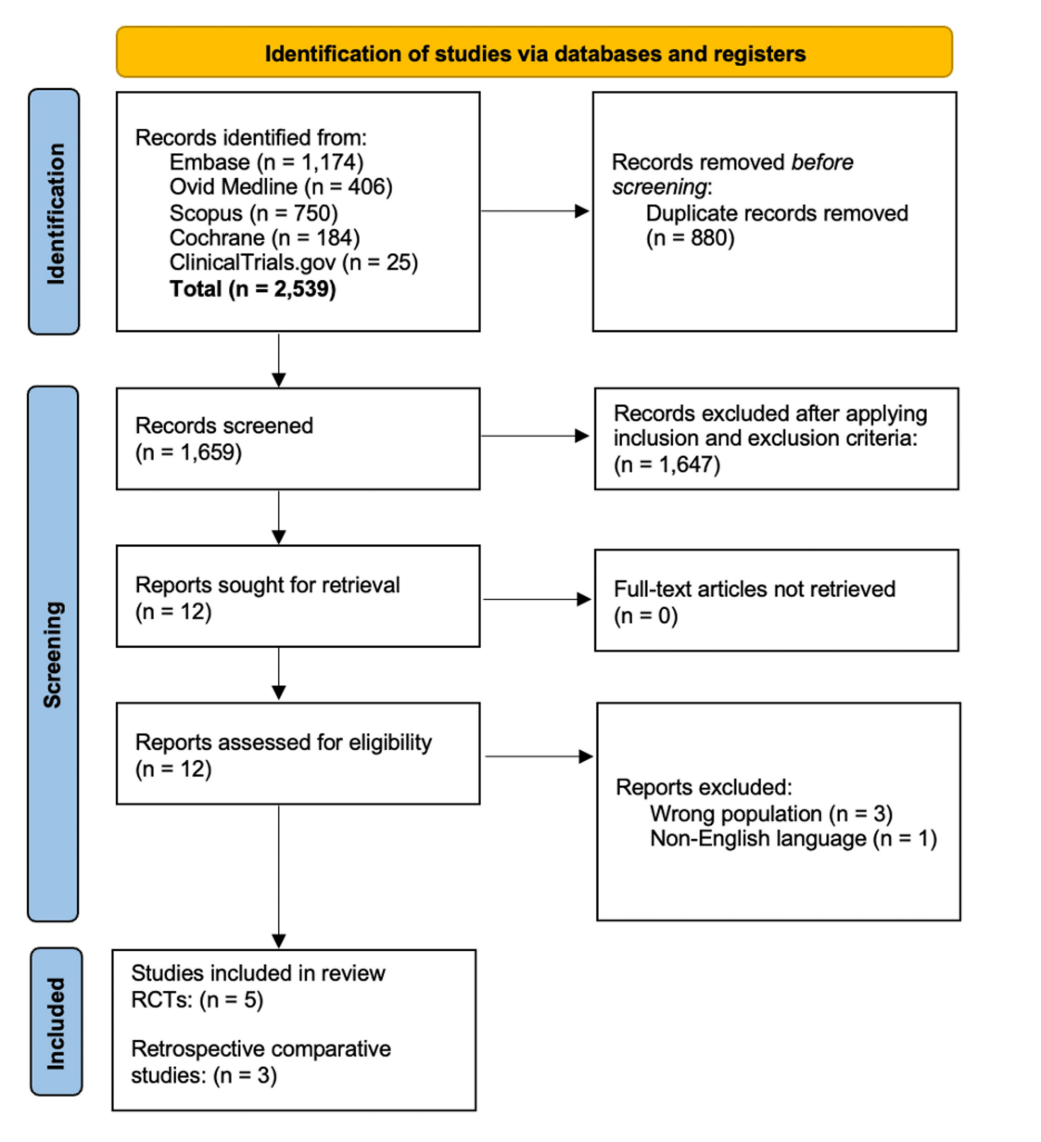
PRISMA scoping review flow diagram outlining the selection process PRISMA: Preferred Reporting Items for Systematic Reviews and Meta-Analyses; RCT: randomized controlled trial

The initial search strategy identified 2,127 records and 286 new results were found in the updated search (2,539 records). After excluding duplicates (n = 880), 1,659 unique articles were independently screened by title and abstract and assessed for eligibility. An additional 1,647 records were excluded through title and abstract screening. Twelve full-text articles were retrieved for further evaluation. Of these, four were excluded for the following reasons: wrong population (n = 3) and non-English language (n = 1). Overall, eight articles met the inclusion criteria. Figure 1 illustrates a PRISMA diagram outlining the literature search and study selection process.

Study Characteristics

The characteristics of the included studies in PICO (population, intervention, comparison, outcome) format are listed in Table 1. Five RCTs [26-30] and three retrospective comparative studies [31-33] met the inclusion criteria for this review. The number of study participants ranged from 50 [28] to 122 [33], totaling 815 patients. All studies compared UG ESIs (n = 387) against FG ESIs (n = 428).

**Table 1:** PICOs of included studies RCT: randomized controlled trials; VNS: Verbal Numeric Scale; VAS: Visual Analogue Scale; NDI: Neck Disability Index; ODI: Oswestry Disability Index; PICO: population, intervention, comparison, outcome

Study (year)	Country	Study type	Population	Intervention	Intervention group demographics (mean age, males : females)	Comparison	Comparison group demographics (mean age, males : females)	Stated outcome/findings
Park et al. (2013) [26]	Republic of Korea	RCT	110 patients	Ultrasound-guidance (n = 55)	(57.27 ± 10.11, 16 : 39)	Fluoroscopy-guidance (n = 55)	(58.47 ± 9.22, 24 : 31)	VNS and ODI significantly improved with continued treatment effects two and 12 weeks after the procedure in both groups. Significant differences were not observed between the two groups.
Jee et al. (2013) [27]	Republic of Korea	RCT	120 patients	Ultrasound-guidance (n = 55)	(56.69 ± 9.32, 23 : 32)	Fluoroscopy-guidance (n = 55)	(57.76 ± 9.56, 24 : 31)	VNS and NDI significantly improved with continued treatment effects two and 12 weeks after the procedure in both groups. Significant differences were not observed between the two groups. Five cases of intravascular injections were observed only in the fluoroscopy-guided group without significant differences between the groups. (p > 0.05).
Park et al. (2015) [31]	Republic of Korea	Retrospective comparative study	110 patients	Ultrasound-guidance (n = 58)	(57.9 ± 9.9, 38 :20)	Fluoroscopy-guidance (n = 52)	(56.3 ± 9.7, 17 : 35)	VNS and ODI significantly improved in both groups at the 3-, 6-, and 12-month post-injection time points. No statistical differences were observed between both groups.
Hazra et al. (2016) [28]	India	RCT	50 patients	Ultrasound-guidance (n = 25)	(44.48 ± 6.48, 11 : 14)	Fluoroscopy-guidance (n = 25)	(41.88 ± 8.05, 9 : 16)	VNS and ODI significantly improved in both groups at the 2-week, 1-month, and 2-month post-injection time points. No statistical differences were observed between both groups. Needle-placement time was less using ultrasound guidance as compared to fluoroscopy guidance (119 ± 7.66 vs. 222.28 ± 29.65 s, respectively, p < 0.001).
Yang et al. (2016) [29]	China	RCT	80 patients	Ultrasound-guidance (n = 40)	(58 ± 9, 16 : 24)	Fluoroscopy-guidance (n = 40)	(57 ± 10, 19: 21)	VAS significantly improved in both groups at the 30-minute, 1-week, and 1-month post-injection time points. No statistical differences were observed between both groups. Operation time was less using ultrasound guidance as compared to fluoroscopy guidance (518 ± 103 vs. 929 ± 228 s, respectively, p < 0.05).
Park et al. (2019) [32]	Republic of Korea	Retrospective comparative study	112 patients	Ultrasound-guidance (n = 51)	(53.5 ± 10.9, 39 : 12)	Fluoroscopy-guidance (n = 61)	(53.5 ± 10.8, 18 : 43)	VNS and NDI significantly improved in both groups at the 1-month, 3-month, and 6-month post-injection time points. No statistical differences were observed between both groups. Five cases of intravascular injections were observed only in the fluoroscopy-guided group without significant differences between the groups.
Elashmawy et al. (2020) [30]	Egypt	RCT	121 patients	Ultrasound-guidance (n = 59)	(42.53 ± 10.30, 25 : 34)	Fluoroscopy-guidance (n = 62)	(42.69 ± 10.48, 26 : 36)	VAS, ODI, straight leg raising, and modified Schober test significantly improved in both groups at the 1-month and 3-month post-injection time points. No statistical differences were observed between both groups.
Jang et al. (2020) [33]	Republic of Korea	Retrospective comparative study	122 patients	Ultrasound-guidance (n = 44)	(52.9 ± 11.9, 12 : 32)	Fluoroscopy-guidance (n = 78)	Interlaminar: (54.8 ± 10.3, 14: 27) Transforaminal: (54.8 ± 10.3, 13 : 24)	VNS and NDI significantly improved in both groups at the 1-month, 3-month, and 6-month post-injection time points. No statistical differences were observed between both groups. Intravascular contrast spread was noted during injection in nine patients who received fluoroscopy-guided injections.

Risk-of-Bias Assessment

**Figure 2:**
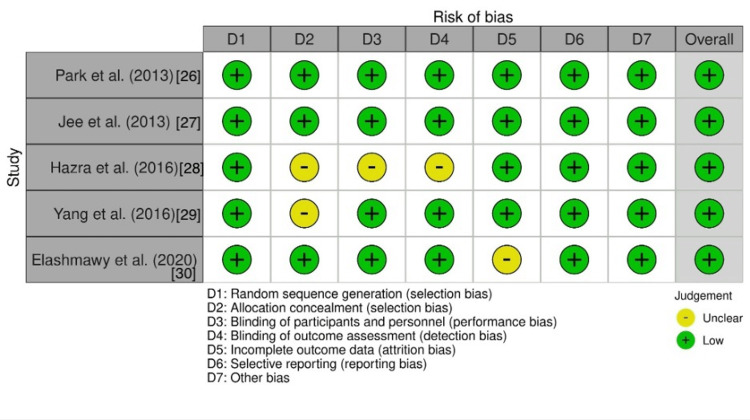
Risk of bias of included RCTs RCT: randomized control trial

The quality of the included RCTs was appraised according to the Cochrane reviewers’ handbook [34]. All five of the included RCTs outlined specific randomization techniques which the authors of this review considered to be at low risk of bias [26-30]. Four of the five included RCTs described blinding techniques during data analysis and evaluation of treatment success [26,27,29,30]. None of the RCTs commented on patient blinding during the administration of treatment as blinding the provider to the imaging modality (ultrasound vs. fluoroscopy) is not feasible. All five RCTs included in this study showed comparable baseline data between the control and treatment groups. Figure 2 shows an illustration of the risk of bias in the included RCT studies (details in Appendix B).

Reduction in Pain and Symptoms

All included studies reported an outcome measure related to the measurement of pain or symptoms of radiculopathy. These outcome measures included the Verbal Numeric Scale (VNS) [26-28,31-33], the Neck Disability Index (NDI) [27,32,33], the Oswestry Disability Index (ODI) [26,28,30,31], the Visual Analogue Scale (VAS) [29,30], straight-leg raising [30], and the modified Schober test [30]. All studies reported a significant improvement in the outcome measures used, with no significant difference between UG and FG ESIs.

Length of Procedure

Two RCTs measured the time required for proper placement of the needle [28,29]. Both studies reported that the total operation time and the time required for proper needle placement were significantly shorter in the UG group as compared to the FG group.

Complications

None of the included studies reported significant adverse effects of ESIs in either the UG or FG groups. However, it appears that FG ESIs may lead to an increased risk of intravascular injection. Park et al. reported two cases of intravascular injection in the FG group with none in the UG group [26]. Jee et al. reported five cases of intravascular injection in the FG group with none in the UG group [27]. Similarly, Jang et al. reported nine cases of intravascular injection in the FG group with none in the UG group [33].

Discussion

This systematic scoping review characterized the evidence regarding the efficacy and safety of UG and FG ESIs for radicular back pain and identified areas where further research is needed to strengthen the evidence base and guide clinical practice. ESIs have traditionally been performed under fluoroscopic guidance [14]. However, healthcare providers are receiving earlier exposure and better training in the use of ultrasound imaging, and the incorporation of ultrasound into the delivery of ESIs has the potential to save the healthcare industry and patients significant financial costs [29,35]. Specifically, the data suggest that UG ESIs are comparable to FG ESIs in terms of pain relief and recovery of motor function. All of the included studies reported no significant difference between the UG and FG groups in various pain and disability assessment outcome measures (VNS, VAS, NDI, ODI, etc.).

Additionally, the two studies that assessed the length of the procedure between the two groups found that ultrasound guidance resulted in a shorter procedure time and faster placement of the needle in the proper position [28,29]. The differences were significant in both cases, suggesting that ultrasound guidance can provide a benefit to both providers and patients by facilitating a faster procedure.

Although no significant adverse events were reported in the included studies, it is notable that three studies found cases of intravascular injection in the FG group, and no cases in the UG group [26,27,33]. This is likely due to the advantage that ultrasound imaging provides of direct, real-time visualization of soft tissue structures and bony surfaces, facilitating needle manipulation and avoidance of vessels and soft tissue structures [14,15].

Overall, the use of ultrasound appears to provide significant advantages over fluoroscopy such as reducing the length of the procedure, reducing exposure to ionizing radiation [36], and facilitating the avoidance of intravascular injections by allowing for real-time visualization of needle placement, all while providing comparable efficacy in terms of pain relief and recovery of motor function.

Strengths and Limitations

This was the first review to explore the literature examining the efficacy and safety of UG versus FG ESIs for the management of radicular back pain. The study was strengthened by the systemic searches of the literature using large bibliometric databases. This scoping review has several limitations. Only English-language articles were included, which may have resulted in the exclusion of significant studies published in other languages. Additionally, non-comparative studies were not included which may have limited the identification of all relevant studies using UG ESIs.

## Conclusions

By focusing on the efficacy and safety of ultrasound guidance (UG) compared to fluoroscopic guidance (FG) epidural steroid injections (ESIs), this scoping review has highlighted the potential advantages that ultrasound can provide, such as real-time visualization of needles and the avoidance of ionizing radiation. Future studies should focus not only on assessing efficacy and complication rates between the two imaging modalities, but also on long-term outcomes, cost-effectiveness, and the impact of UG ESIs on patient satisfaction and quality of life.

## Appendices

Appendix A

Full Search Strategies

Embase

Date Searched: 4/13/2022

Applied Database Supplied Limits: none

Number of Results:  1,009

Search update: 1/2/2024

Results: 1,174 (165)

Full Search Strategy:

('radicular pain'/exp OR 'radiculopathy'/exp OR ((Radicular OR ‘nerve root') NEAR/3 (pain OR disease OR neuralgia OR neuropathy)):ti,ab,kw OR (radiculalgia OR radiculopathy OR polyradiculopathy):ti,ab,kw) AND (('ultrasound'/exp OR 'echography'/exp OR 'color Doppler flowmetry'/exp OR (‘Ultrasound-guided’ OR ‘US-guided’ OR ‘ultrasound guided’ OR ultrasound OR phonophoresis OR sonication OR sonification OR ‘ultra sound’ OR ultrashell OR ultrasonic* OR ultrasonography OR echography OR doptone OR echogram OR echoscopy OR echosound OR sonogram OR sonography OR ultrasonogram OR ultrasonographic):ti,ab,kw OR (color NEAR/3 (flowmetry OR Doppler OR ultrasonography)):ti,ab,kw) OR ('fluoroscopy'/exp OR (‘fluoroscopy-guided’ OR ‘FL-guided’ OR fluoroscopy OR fluorophotography OR photofluoroscopy OR radiofluoroscopy OR roentgenfluoroscopy):ti,ab,kw OR ((fluorescence OR fluorescent) NEAR/3 (radiation OR scanning)):ti,ab,kw)) AND ('epidural drug administration'/exp OR 'nerve root block'/exp OR 'epidural anesthesia'/exp OR 'selective nerve root block'/exp OR 'nerve root'/exp OR 'epidural steroid injection'/exp OR epidural:ti,ab,kw OR ((dural OR extradural OR peridural OR nerve OR transforaminal) NEAR/2 (block* OR anaesthesia OR anesthesia OR injection*)):ti,ab,kw OR (‘nerve root’ OR SNRB OR CESI OR ‘nerve radix’ OR ‘neutral root’ OR ‘radix nervi’):ti,ab,kw)

Ovid Medline

Date Searched: 4/13/2021 Applied Database Supplied Limits: none Number of Results:  358

Search update: 1/2/2024

Results: 406 (48) Full Search Strategy:

(exp Radiculopathy/ OR ((Radicular OR nerve root) ADJ3 (pain OR disease OR neuralgia OR neuropathy)).ti,ab,kf. OR (radiculalgia OR radiculopathy OR polyradiculopathy).ti,ab,kf.) AND ((exp Ultrasonography, Interventional/ OR exp Ultrasonography, Doppler, Color/ OR (Ultrasound-guided OR US-guided OR ultrasound guided OR ultrasound OR phonophoresis OR sonication OR sonification OR ultra sound OR ultrashell OR ultrasonic* OR ultrasonography OR echography OR doptone OR echogram OR echoscopy OR echosound OR sonogram OR sonography OR ultrasonogram OR ultrasonographic).ti,ab,kf. OR (color ADJ3 (flowmetry OR Doppler OR ultrasonography)).ti,ab,kf.) OR (exp Fluoroscopy/ OR (fluoroscopy-guided OR FL-guided OR fluoroscopy OR fluorophotography OR photofluoroscopy OR radiofluoroscopy OR roentgenfluoroscopy).ti,ab,kf. OR ((fluorescence OR fluorescent) ADJ3 (radiation OR scanning)).ti,ab,kf.)) AND (exp Anesthesia, Epidural/ OR exp Analgesia, Epidural/ OR exp Injections, Epidural/ OR exp Spinal Nerve Roots/ OR epidural.ti,ab,kf. OR ((dural OR extradural OR peridural OR nerve OR transforaminal) ADJ2 (block* OR anaesthesia OR anesthesia OR injection*)).ti,ab,kf. OR (nerve root OR SNRB OR CESI OR nerve radix OR neutral root OR radix nervi).ti,ab,kf.)

Scopus

Date Searched: 4/13/2022 Applied Database Supplied Limits: none Number of Results:  617

Search update: 1/2/2024

Results: 750 (133) Full Search Strategy:

((TITLE-ABS-KEY((Radicular OR “nerve root”) W/3 (pain OR disease OR neuralgia OR neuropathy))) OR (TITLE-ABS-KEY(radiculalgia OR radiculopathy OR polyradiculopathy))) AND (((TITLE-ABS-KEY(“Ultrasound-guided” OR “US-guided” OR “ultrasound guided” OR ultrasound OR phonophoresis OR sonication OR sonification OR “ultra sound” OR ultrashell OR ultrasonic* OR ultrasonography OR echography OR doptone OR echogram OR echoscopy OR echosound OR sonogram OR sonography OR ultrasonogram OR ultrasonographic)) OR (TITLE-ABS-KEY(color W/3 (flowmetry OR Doppler OR ultrasonography)))) OR ((TITLE-ABS-KEY(“fluoroscopy-guided” OR “FL-guided” OR fluoroscopy OR fluorophotography OR photofluoroscopy OR radiofluoroscopy OR roentgenfluoroscopy)) OR (TITLE-ABS-KEY((fluorescence OR fluorescent) W/3 (radiation OR scanning))))) AND ((TITLE-ABS-KEY((dural OR extradural OR peridural OR nerve OR transforaminal) W/2 (block* OR anaesthesia OR anesthesia OR injection*))) OR (TITLE-ABS-KEY(“nerve root” OR SNRB OR CESI OR “nerve radix” OR “neutral root” OR “radix nervi”)))

The Cochrane Library

Date Searched: 4/13/2022 Applied Database Supplied Limits: none Number of Results

CENTRAL: 133

CDSR: 0

Search update: 1/2/2024

Results

CENTRAL: 184 (51)

CDSR: 0

Full Search Strategy:

([mh “radicular pain”] OR [mh “radiculopathy”] OR ((Radicular OR “nerve root”) NEAR/3 (pain OR disease OR neuralgia OR neuropathy)):ti,ab,kw OR (radiculalgia OR radiculopathy OR polyradiculopathy):ti,ab,kw) AND (([mh “ultrasound”] OR [mh “echography”] OR [mh “color Doppler flowmetry”] OR (“Ultrasound guided” OR “US guided” OR “ultrasound guided” OR ultrasound OR phonophoresis OR sonication OR sonification OR “ultra sound” OR ultrashell OR ultrasonic* OR ultrasonography OR echography OR doptone OR echogram OR echoscopy OR echosound OR sonogram OR sonography OR ultrasonogram OR ultrasonographic):ti,ab,kw OR (color NEAR/3 (flowmetry OR Doppler OR ultrasonography)):ti,ab,kw) OR ([mh “fluoroscopy”] OR (“fluoroscopy guided” OR “FL guided” OR fluoroscopy OR fluorophotography OR photofluoroscopy OR radiofluoroscopy OR roentgenfluoroscopy):ti,ab,kw OR ((fluorescence OR fluorescent) NEAR/3 (radiation OR scanning)):ti,ab,kw)) AND ([mh “epidural drug administration”] OR [mh “nerve root block”] OR [mh “epidural anesthesia”] OR [mh “selective nerve root block”] OR [mh “nerve root”] OR [mh “epidural steroid injection”] OR epidural:ti,ab,kw OR ((dural OR extradural OR peridural OR nerve OR transforaminal) NEAR/2 (block* OR anaesthesia OR anesthesia OR injection*)):ti,ab,kw OR (“nerve root” OR SNRB OR CESI OR “nerve radix” OR “neutral root” OR “radix nervi”):ti,ab,kw)

ClinicalTrials.gov

Date Searched: 4/13/2022

Number of Results:  10 Search update: 1/2/2024

Results: 25 (15)

Full Search Strategy:

“radicular pain” AND (“ultrasound guided” OR “fluoroscopy guided”) AND epidural

Appendix B

Risk of Bias in the Included RCTs

**Table 2:** Risk-of-bias assessment Park et al., 2013 [26] US: ultrasound; FL: fluoroscopy

Bias	Authors’ judgment	Support for judgment
Random sequence generation (selection bias)	Low risk	“The participants were randomly assigned to one of two groups using a computer-generated randomization table: US and FL groups.”
Allocation concealment (selection bias)	Low risk	“To avoid research bias, pain, function, and satisfaction scores were evaluated by a third person or a researcher totally blinded to the entire procedure of this study or the methods used.”
Blinding of participants and personnel (performance bias)	Low risk	“To avoid research bias, pain, function, and satisfaction scores were evaluated by a third person or a researcher totally blinded to the entire procedure of this study or the methods used.”
Blinding of outcome assessment (detection bias)	Low risk	“A radiologist with more than 7 yrs of experience in musculoskeletal image, who was blinded to the injection methods, judged all radiographs simultaneously by consensus.”
Incomplete outcome data (attrition bias)	Low risk	“Four patients in the US group and two patients in the FL group were excluded for no improvement or worsening of pain during the first follow-up period, after the first injections. Three patients in the FL group were excluded, with two patients with peripheral injections (shoulder and knee) and one patient having been administered medication other than acetaminophen. One patient in the US group with a peripheral injection (hip) was also excluded.” No patients were lost to follow-up.
Selective reporting (reporting bias)	Low risk	Not evidence of selective reporting.
Other bias	Low risk	No evidence of other bias.

**Table 3:** Risk-of-bias assessment Jee et al., 2013 [27] US: ultrasound; FL: fluoroscopy

Bias	Authors’ judgment	Support for judgment
Random sequence generation (selection bias)	Low risk	“Participants were randomly assigned into one of two groups using a computer-generated randomization table: US and FL groups.”
Allocation concealment (selection bias)	Low risk	“It is difficult to conduct a double-blinded control study with the non-traditional modalities such as ultrasound or fluoroscopy. However, we tried to minimize the research bias by evaluating the pain, function, and satisfactory score by a researcher unfamiliar with the injection methods. “
Blinding of participants and personnel (performance bias)	Low risk	“All images were assessed by a radiologist, blinded to the injection methods, for all radiographic findings.”
Blinding of outcome assessment (detection bias)	Low risk	“Data collection and the analysis on the treatment effects were performed by a researcher blinded to the procedures.”
Incomplete outcome data (attrition bias)	Low risk	“Three US and five FL patients were excluded for no improvement or worsening of pain during the first follow-up period, after the first injections. Two patients in US were excluded: one had peripheral injections in the shoulder, and one administered medication other than acetaminophen during the study period.” No patients were lost to follow-up.
Selective reporting (reporting bias)	Low risk	No evidence of selective reporting.
Other bias	Low risk	No evidence of other bias.

**Table 4:** Risk-of-bias assessment Hazra et al., 2016 [28]

Bias	Authors’ judgment	Support for judgment
Random sequence generation (selection bias)	Low risk	“Randomisation was done using ‘coin flip’ method. A coin was flipped for every two consecutive patients; the first patient was allocated to Group U on getting ‘head’ or to Group F on getting ‘tail’. The next of this set was allocated to the other group.”
Allocation concealment (selection bias)	Unclear risk	Authors did not comment on allocation concealment.
Blinding of participants and personnel (performance bias)	Unclear risk	Authors did not comment on blinding.
Blinding of outcome assessment (detection bias)	Unclear risk	Authors did not comment on blinding.
Incomplete outcome data (attrition bias)	Low risk	No losses to follow-up reported.
Selective reporting (reporting bias)	Low risk	No evidence of selective reporting.
Other bias	Low risk	No evidence of other bias.

**Table 5:** Risk-of-bias assessment Yang et al., 2016 [29] US: ultrasound; FL: fluoroscopy

Bias	Authors’ judgment	Support for judgment
Random sequence generation (selection bias)	Low risk	“Patients were then randomly divided into the FL-guided group or the US-guided group according to a random number table.”
Allocation concealment (selection bias)	Unclear risk	The authors did not comment on allocation concealment.
Blinding of participants and personnel (performance bias)	Low risk	“If the 2 physicians agreed that the US-guided operation was successful, then the ratio of operation success in the US group was confirmed using FL by another physician who was blinded to the operation and had several years of experience.”
Blinding of outcome assessment (detection bias)	Low risk	“Complications, including infection, aggravation of the pain, numbness, headache, and any allergic reactions were also recorded by a physician who was blinded to the operation.”
Incomplete outcome data (attrition bias)	Low risk	No losses to follow-up reported.
Selective reporting (reporting bias)	Low risk	No evidence of selective reporting.
Other bias	Low risk	No evidence of other bias.
